# A systematic review of Health Technology Assessment tools in sub-Saharan Africa: methodological issues and implications

**DOI:** 10.1186/1478-4505-12-66

**Published:** 2014-12-02

**Authors:** Christine Kriza, Jill Hanass-Hancock, Emmanuel Ankrah Odame, Nicola Deghaye, Rashid Aman, Philip Wahlster, Mayra Marin, Nicodemus Gebe, Willis Akhwale, Isabelle Wachsmuth, Peter L Kolominsky-Rabas

**Affiliations:** Interdisciplinary Centre for Health Technology Assessment (HTA) and Public Health (IZPH), University of Erlangen-Nürnberg, Schwabachanlage 6, Erlangen, 91054 Germany; Health Economics and HIV/AIDS Research Division (HEARD), University of KwaZulu-Natal, Westville Campus, J block, Level 4, University Road, Private Bag X54001, Durban, 4041 South Africa; Ghana Ministry of Health, MB 44 Accra, Ghana; Centre for Research in Therapeutic Sciences (CREATES), Strathmore University, Ole Sangale Road, 59857-00200 Nairobi, Kenya; Ghana Ministry of Health, M44 Accra, Ghana; Kenya Ministry of Public Health and Sanitation, Kenyatta Hospital Grounds, PO Box 19982-00202, Nairobi, Kenya; World Health Organization, 20 Avenue Appia, Geneva, 1211 Switzerland

**Keywords:** Health services, Health systems, Health systems research

## Abstract

**Background:**

Health technology assessment (HTA) is mostly used in the context of high- and middle-income countries. Many “resource-poor” settings, which have the greatest need for critical assessment of health technology, have a limited basis for making evidence-based choices. This can lead to inappropriate use of technologies, a problem that could be addressed by HTA that enables the efficient use of resources, which is especially crucial in such settings. There is a lack of clarity about which HTA tools should be used in these settings. This research aims to provide an overview of proposed HTA tools for “resource-poor” settings with a specific focus on sub-Saharan Africa (SSA).

**Methodology:**

A systematic review was conducted using basic steps from the PRISMA guidelines. Studies that described HTA tools applicable for “resource-limited” settings were identified and critically appraised. Only papers published between 2003 and 2013 were included. The identified tools were assessed according to a checklist with methodological criteria.

**Results:**

Six appropriate tools that are applicable in the SSA setting and cover methodological robustness and ease of use were included in the review. Several tools fulfil these criteria, such as the KNOW ESSENTIALS tool, Mini-HTA tool, and Multi-Criteria Decision Analysis but their application in the SSA context remains limited. The WHO CHOICE method is a standardized decision making tool for choosing interventions but is limited to their cost-effectiveness. Most evaluation of health technology in SSA focuses on priority setting. There is a lack of HTA tools that can be used for the systematic assessment of technology in the SSA context.

**Conclusions:**

An appropriate HTA tool for “resource-constrained” settings, and especially SSA, should address all important criteria of decision making. By combining the two most promising tools, KNOW ESSENTIALS and Multi-Criteria Decision Analysis, appropriate analysis of evidence with a robust and flexible methodology could be applied for the SSA setting.

## Background

Health technology assessment (HTA) is a multidisciplinary field of policy analysis that examines the medical, economic, social, and ethical implications of the incremental value, diffusion, and use of a medical technology in health care [1]. Currently, HTA is mostly used in the context of developed countries. Several methodologies exist for “resource-poor” settings but implementation of HTA and transparent use in most African countries is still limited [2]. In addition, some methodological aspects of HTA do not fit into the setting of developing countries and need to be adapted appropriately according to specific needs [2]. Especially in “resource-constrained” settings, the need for the systematic evaluation of health technology and of the available alternative technologies has never been greater [3, 4]. HTA is performed in order to improve the quality of health care and ensure good value for money investments in any setting. It is because of this that HTA should form the basis for health technology policies especially in “resource-poor” settings with limited health sector budgets.

The current lack of HTA in sub-Sahara Africa (SSA) can be attributed to the lack of capacity to undertake HTA. Even though countries like South Africa currently employ HTA to a small degree and have begun the process of forming a HTA mechanism, the lack of capacity coupled with a weak health system capacity to implement interventions are contributing to widespread implementation of HTA. Another problematic issue is the limitation of high-quality data availability and lack of research evidence, especially in the context of “resource-limited” health systems like the assessment of health states [5, 6]. This reduces the ability for “resource-poor” settings to implement rigorous HTA practices. In addition, “resource-limited” countries have few resources to support HTA, which then undermines the ability of HTA to utilize appropriate incentives (that would promote more efficient management of resources.

Incentives for HTA in resource-limited settings:

**What are the incentives for resource-limited countries to develop HTA agencies?**

**1 – Providing international guidelines and model essential medicines lists as well guidance on good and ethical governance practices specifically in liaison with ethic review committee and bioethics committee**

Low-income countries with very limited capacity and countries that face major challenges of lack of transparency in decision-making are likely to face difficulty implementing HTA for medicines effectively. Use of international guidelines and model essential medicines lists can help such countries to incorporate HTA in their policies.

**2 – Complement HTA with evidence-based guidelines**

Policies to encourage doctors to prescribe formulary medicines and follow evidence-based guidelines are needed to complement HTA.

**3 – Strengthening capacity in HTA before to set-up HTA agencies through national capacity building workshops specifically for decision makers and health professionals**

Capacity for HTA in resource limited settings should be established early and supported; prerequisites and barriers are extensive but not insurmountable and must be considered as HTA processes are developed.

Decisions in many “resource-poor” settings can easily be influenced by past experience without an evidence base and by preferences of donor agencies and lobbying pressure for new technologies, for example from commercial organisations or global funding and donor organizations [2]. This can lead to the use of technologies which do not address health needs and in effect contribute to the inefficient use of resources [7]. Decisions made in this context often do not reflect local values and evidence based local information on clinical and cost effectiveness [2]. As a result of this, many resources are disproportionately allocated or wasted. The need for HTA becomes more prominent when the need for collective decision making for the good of the whole is necessary. It also becomes apparent that HTA in “resource-poor” settings cannot be addressed the same as it is in high-income countries [8]. Equity and equality considerations are different from these in developed countries. These include more important social issues like poverty reduction [9].

There is a clear need for HTA use in “resource-limited” settings, and SSA countries in particular, as it is these countries especially that cannot afford to waste resources. Not only would HTA highlight health technologies that would be too costly relative to their benefits, but would also identify potentially harmful and ineffective technologies. In light of a higher percentage of insured patients stemming from advanced economic development and the potential to purchase more and more complex, and expensive, health technologies, the introduction of HTA into public health policies becomes more evident. The WHO resolution on “Health Intervention and Technology Assessment in Support of Universal Health Coverage” passed on 24 May 2014 by the World Health Assembly provides an important mandate for SSA countries to accelerate their HTA efforts.

### Objectives

The research objectives of this systematic review are to provide an overview of HTA tools used in “resource-constrained” settings, with a specific focus on the assessment on SSA. It was chosen as the focus setting because it is the region with the least covered area by HTA practices and also the most “resource-constrained” setting. Another objective is to determine how many of the appropriate HTA tools identified in this review were applied for the assessment of medical devices.

## Methods

### Search method

The PRISMA guidelines for conducting systematic reviews were followed [10]. Within the study the following databases were searched: PubMed, Science Direct, Scopus, Ebscoh, and EconLit alongside the journals *Health Policy and Planning*, *Cost-Effectiveness and Resource Allocation*, and the WHO Bulletin (see Appendix for detailed search string). To ensure optimal coverage, additional articles were found within the reference section of retrieved articles and through citation snowballing by undertaking wider searches by author name for those appearing as key publishers in the area. Additionally, the web pages of the WHO and the World Bank were searched manually. The search was limited to articles published within the 2003 and 2013 timeframe and excluded non-empirical studies or those that did not focus on a HTA tool.

### Selection of manuscripts and data extraction

Articles that met the inclusion criteria of an evidence-based HTA tool appropriate for the use in the SSA context were retrieved and examined more closely. The quality of research papers was evaluated according to adequate description of the theoretical framework, background, and methodology [11].

For those papers that fulfilled the criteria for quality, data was extracted according to the following content: date published, study funding source, possible conflicts of interest, study objectives, target population, application of tool, site/setting, study focus, HTA tool proposed or approach used in the paper, description of tool or approach, stand alone or support tool, aspects of clinical effectiveness, costs and contextual issues addressed by tool or approach, all stakeholders involved, literature search incorporated, results of implementation, and focus on medical technology/intervention.

Each study was described by addressing the criteria in the data extraction form (Table 1).

**Table 1:** **Extraction form for study characteristics**

Author(s)	Mathew	Abaza and Tawfik	Hutubessy et al.	Miot et al.	Govender et al.	Ueffing et al.
**Study name**	KNOW ESSENTIALS: A tool for informed decisions in the absence of formal HTA systems	Appropriate medical technologies for developing countries: application to cardiovascular disorders	Generalized cost-effectiveness analysis for national-level priority-setting in the health sector	Field testing of a multicriteria decision analysis (MCDA) framework for coverage of a screening test for cervical cancer in South Africa	Purchasing of medical equipment in public hospitals: the mini-HTA tool	Equity-oriented toolkit for health technology assessment and knowledge translation: application to scaling up of training and education for health workers
**Published date**	2011	2008	2003	2012	2011	2009
**Study funding source(s)**	None	Not stated	Not stated	Not stated	Not stated	Not stated
**Possible conflicts of interest**	None	Not stated	None	None	None	Three authors expressed competing interests because of their affiliation with the WHO
**Objective**	Describes a tool, “KNOW ESSENTIALS” that includes current best evidence on health technologies, incorporates relevant contextual issues, and is objective, reproducible, transparent, and affordable	Provide an acquisition methodology by which healthcare providers can minimize the underutilization of medical devices to be purchased when certain diseases are to be dealt with and allow non-technical personnel to make correct and appropriate acquisitions	Outline the process by which country level decision makers and programme managers can carry out their own context-specific analysis of the relative cost-effectiveness of interventions for reducing leading causes of national disease burden using cost-effective analysis (CEA) information from the WHO-CHOICE project	Field testing of the EVIDEM framework for decision-making on a screening test by a private health plan in South Africa	Adapt and use the Danish Centre for Evaluation and Health Technology Assessment (DACEHTA) mini-HTA tool to assess past decisions made by South African hospital managers, as applied to selected medical devices	Propose a toolkit for decision-makers to scale up training and education of health workers
**Target population**	Settings lacking formal HTA systems	Developing countries	Low-income settings	Low-resource settings	South African hospitals	Economically disadvantaged areas
**Was tool/approach applied?**	Yes, pilot tested	Yes, pilot tested	No	Yes	Yes	No
**Site/setting**	Africa (hypothetical)	Not stated	n/a	South Africa	South Africa	n/a
**Study focus**	Favourability of artemisinin-based treatment for severe or complicated malaria in children using KNOW ESSENTIALS	Cardiovascular disorder equipment purchases with support database software	n/a	Cervical cancer screening decision-making	Decision support checklist for hospital managers to inform decisions about the acquisition of health technologies (drugs, devices, and other health interventions)	n/a
**Expressed need for tool or approach**	Lack of formal HTA or low level of application in developing countries; decisions are highly subjective and expert based rather than research based	Lack of HTA and the recognition for the need of HTA in developing countries	Shortage of technical expertise and health service capacity to utilize CEA information	Need for transparency and greater access to evidence through a systematic and explicit process	Existence of management information gaps in South African public hospitals and need for a customized tool to support decision makers in medical device management	Need to address the shortage of health workers which are considered part of health care resources
**HTA tool proposed and designed to be applied for specific medical technologies**	KNOW ESSENTIALS	Decision support database software	n/a	n/a	Mini-HTA tool/ hospital-based HTA tool	Equity-Oriented Toolkit (EOT)
**HTA approach used in the paper (if new tool not proposed)**	n/a	n/a	Use of CEA information from the WHO-CHOICE project, generalized CEA	Use of the EVIDEM (Evidence and Value: Impact on DEcision Making) framework, brings together HTA and MCDA	n/a	n/a
**Applied in the context of medical devices?**	No	Yes	No	Yes	Yes	No
**Description of tool or approach**	Elements addressing different aspects of HTA divided into background issues (KN, O, W), essential criteria (E, S, S, E), and other criteria (N, T, I, A, L, S). Critical appraisal through a systematic review process with meta-analysis, or using other clearly defined search strategies with justification. Colour coding of elements when available information favours (green)/does not favour (red) the medical technology, or (yellow) if available information is insufficient to classify green or red. For last six elements, not applicable (white) code available. If any Background Issues coded red, health technology is rejected, if not it proceeds to Essential Criteria. If any coded red, health technology is rejected, if not proceeds to Other Criteria. If majority red, health technology is rejected, considered favourably if mostly green, deferred if mostly yellow	Database constructed by compiling data about cardiovascular disease equipment specifications. Database comprised of three main forms. First form enables user to select criteria on the disease, the brand, equipment type, and non-diagnostic features. Second form lists relevant equipment according to selected criteria. Equipment is ordered by a priority scheme depending on total number of diagnostic features, number of unused diagnostic features, and number of diagnostic features for arrhythmias labelled and highlights less recommended equipment based on these features. The third form allows user to examine selected equipment more closely	Generalized CEA identifies current allocative inefficiencies as well as opportunities presented by new interventions and presents it in a way that can be translated across settings by i) evaluating the costs and health benefits of a set of related interventions, singly and in combination, with the “null scenario”; ii) using CEA results to classify interventions into those that are very cost-effective, cost-ineffective, and somewhere in between rather than using the traditional league table approach	After a literature review and input form a clinical committee of the health plan, a HTA report on liquid based cytology (LBC) for cervical cancer screening was tailored to investigate 14 MCDA inclusion criteria and four contextual criteria (appraised qualitatively) proposed by healthcare funder. The contents of report were tailored to local context. Committee engaged in workshops where members assigned weights to each criterion of the MCDA matrix and scores for LBC for each criterion of the MCDA matrix based on the data of the HTA report. Members then assigned qualitative impact of system-related criteria on the appraisal. Adoptability and utility of framework were explored through a post-testing survey	Adaptation of the DACEHTA tool, which is separated into the following sections: introduction, technology, patient, organization, economy. The tool was adapted into the following cluster: patients, technology, economy, and organizational influence. The tool was used as a prospective cross-sectional survey concerning the decision-making process of purchasing medical devices over the past year, administered to 21 hospital managers	Adaptation of the WHO’s Needs-Based Toolkit for Health Technology Assessment that was created to aid health policy makers and planners to allocate resources efficiently, fairly, and effectively. A perspective of “equity” was added in the EOT, based on clinical and population health status. Four major steps: burden of illness, community effectiveness, economic evaluation, and knowledge translation and implementation. Recommendations were given for scaling up education and training of health workers.
**Stand-alone tool or a support tool for existing decision making process?**	Stand alone	Support	Support	Stand alone	Support	Stand alone
**Results of implementation**	Atemisinin-based treatment for severe or complicated malaria in children should be incorporated as the first-line treatment in the National guideline. (hypothetical)	Concluded that this tool would save effort from technical personnel and is friendly enough to be used by non-technical personnel. It would also be a helpful tool for the determination of budget and other non-diagnostic criteria.	n/a	Resulted in a consideration by the health plan to only fund for LBC up to the value of conventional pap smears. A negotiation process was started with the pathology laboratories and the fee for LBC was reduced to an amount which was considered appropriate for full funding; 50% of members felt that EVIDEM improved understanding of the intervention, access to quality assessment of the evidence on the intervention, and consideration of all key elements of the decision; 56% felt it improved transparency of decision making. No member thought it worse than existing process	Study results showed deficiencies for medical technology: no sufficient consideration of risks related to a medical technology or on the impact on staff or costs	n/a

The HTA tool or approach found in each study were critically appraised using a second data extraction form (Table 2) based on proposed criteria for the assessment of HTA activities [12]. These criteria are formed by a series of 15 principles, as described by Drummond et al. [12], which cover the structure of HTA programs, methods of HTA, processes for conduct of HTA, and use of HTAs in decision making. This set of principles was utilized to ensure robustness of the included HTA approaches and tools. The extraction form prompted i) the context and applicability of the described tool (geographical focus, target setting, reasons for use, degree of needed training, and experts); ii) measures for sensitivity and effectiveness (clinical effectiveness, cost effectiveness, context sensitivity); iii) the approach to HTA assessment (type of tool, inclusion of full social perspective, use of available evidence, transparency, generalizability, result focus, ling to decision making), and iv) if the described tool was piloted for medical devices.

**Table 2:** **Extraction form for principles of HTA activities according to Drummond et al.** [12]

Author(s)	Mathew	Abaza and Tawfik	Hutubessy et al.	Miot et al.	Govender et al.	Ueffing et al.
**Study name**	KNOW ESSENTIALS: A tool for informed decisions in the absence of formal HTA systems	Appropriate medical technologies for developing countries: application to cardiovascular disorders	Generalized cost-effectiveness analysis for national-level priority-setting in the health sector	Field testing of a multicriteria decision analysis (MCDA) framework for coverage of a screening test for cervical cancer in South Africa	Purchasing of medical equipment in public hospitals: the mini-HTA tool	Equity-oriented toolkit for health technology assessment and knowledge translation: application to scaling up of training and education for health workers
**Structure of HTA Programs**						
**Are the goal and scope of the HTA explicit and relevant to its use?**	Yes, outcomes of interest are clearly defined at the beginning of process (no detailed scoping document mentioned)	Yes, deals with specific disease and all relevant health technologies (medical devices)	Yes, cost effectiveness analysis of specific health technology	Yes, HTA report clearly outlines the purpose	Yes, the form clearly asks to define medical technology and scope of proposal	Yes, criteria and requirements are clearly defined
**Is it unbiased and transparent?**	Yes, evidence based and steps have clear criteria (no independent party conducting HTA is mentioned)	Yes, systematic and evidence-based software	Yes, evidence based and systematic approach	Yes, evidence based and priorities of stakeholders are clearly defined and addressed	No, the stakeholders working on the form can make subjective assessments	Yes, evidence based and different stakeholders are involved
**Does it include all relevant technologies?**	Yes, takes into account alternatives	Yes, all medical devices dealing with disease and diagnostic procedure of interest are included in database	Yes, the WHO-CHOICE project used includes an extensive database of evidence	Yes, considers alternatives	Yes, considers alternatives	Not stated
**Does a clear system for setting priorities exist?**	Not stated, does not mention priority setting prior to the implementation of tool	Yes, systematic search by software of medical devices most relevant to stakeholder’s preferences	Not stated, does not mention priority setting prior to the implementation of approach	Yes, weights were assigned to criteria of the framework by stakeholders	Not stated, does not mention priority setting prior to the implementation of approach	Yes, includes concepts of needs assessment and priority setting
**Methods of HTA**						
**Does it incorporate appropriate methods for assessing costs and benefits?**	Yes, assesses available evidence to determine costs of technology and providing the technology and its cost-effectiveness. Also assesses effectiveness and safety of technology	No, does not assess cost-effectiveness of health technologies at this moment	Yes, tool determines cost-effectiveness and assesses the benefits and drawbacks of implementing or not implementing the health technology along with combinations of health technologies	Yes, HTA report assesses the economics and various benefits of the intervention	Yes, addresses costs for different stakeholders and assesses the risks and benefits of the health technology	Yes, in its economic evaluation it assesses the benefits and costs as well as the trade-offs between equity and efficiency
**Does it consider a wide range of evidence and outcomes?**	Yes, considers available evidence and long-term outcomes of using or rejecting the health technology	Yes, it incorporates all available information about the medical devices	Yes, with the use of the WHO-CHOICE project databases for evidence and one of its tools, PopMod, for analysing outcomes of using and rejecting health technology (among other scenarios)	Yes, a thorough search for evidence in different databases and other sources is undertaken for each criterion	Yes, a search and quality assessment is undertaken of the available literature	Yes, uses a strong evidence base
**Is a full societal perspective considered?**	Yes, takes into account social issues and interests of different stakeholders	Not stated	Not stated	Not stated	Yes, takes into consideration effects of proposal on other departments in the hospital and the cooperation with other hospitals	Yes, includes both societal and individual determinants
**Does it explicitly characterize uncertainty surrounding estimates?**	Not stated	Not stated	Yes, with the use Monte Carlo League software, an analytical tool, to find the uncertainty around point estimates	Yes, quality of evidence is assessed in the HTA report portion of approach	Yes, the person filling out the form notes the uncertainties that apply to the calculations	Not stated
**Does it consider and address issues of generalizability and transferability?**	Yes, generalizability and transferability of evidence from similar cohorts needs to be justified	Not stated	Yes, the WHO-CHOICE project uses international dollars to be able to make meaningful comparisons and adjustments according to practice settings are made to resulting estimates of generalized CEA	Yes, local costs were used when assessing cost-effectiveness to improve transferability	Not stated	Yes, considers community effectiveness or the “real world” efficacy of an intervention
**Processes for Conducting HTA**						
**Are all key stakeholder groups actively engaged?**	Yes, tool includes or considers stakeholders throughout its process	No, only purchaser of medical devices is actively engaged and consideration of key stakeholders is not stated	No, not all stakeholders addressed	Yes, key stakeholders are included throughout the process	Yes, key stakeholders are included or considered throughout the process	Yes, forms a national planning authority that brings together different stakeholders
**Is all available data actively being sought?**	Yes, all available data is sought and used in decision making table	Yes, all available data about medical devices included is actively sought and regular updates are mentioned	Yes, all available data is sought including contextual data	Yes, all available data is sought during the HTA report process	Yes, all available data is sought and consulting a librarian to ensure quality is advised	Yes, strong evidence base is needed for the implementation of this tool
**Are findings monitored?**	Not stated	Not stated	Not stated	Not stated	Not stated	Not stated
**Use of HTA in Decision-Making**						
**Is it timely?**	Not stated	Yes, it is a system of three forms that can be done very quickly	Not stated	Not stated	Yes, the form takes within 5 to 15 hours to answer (excluding evidence retrieval and assessment)	Not stated
**Are findings communicated appropriately to different decision makers?**	Yes, the Decision-Making Table allows decision makers to see the evidence related to the criteria and become informed of the health technologies being assessed	Not stated	Not stated	Yes, the HTA report allows decision makers to see the evidence related to the criteria and become informed of the health technologies being assessed	Yes, the form gives a clear overview for decision makers	Yes, it has included new advances in knowledge translation
**Is the link between HTA findings and decision-making processes transparent and clearly defined?**	Yes, HTA findings and decision making process are clearly separate	Not stated	Not stated	Yes, HTA findings and decision making process are clearly separate	Yes, the purpose of the mini-HTA is stated to be only part of the basis of a proposal for decision makers	Not stated

Criteria addressing areas particular to SSA were included in the evaluation to assess how adaptable the tool or approach would be for that setting. The criteria included the following: ease of training, flexibility of evidence requirement, economical standing, and local context consideration (Table 3). These criteria have been elaborated in consensus with our Research Consortium Members including representatives from SSA countries.

**Table 3:** **Extraction form for principles for HTA activities in SSA**

Author(s)	Mathew	Abaza and Tawfik	Hutubessy et al.	Miot et al.	Govender et al.	Ueffing et al.
**Study name**	KNOW ESSENTIALS: A tool for informed decisions in the absence of formal HTA systems	Appropriate medical technologies for developing countries: application to cardiovascular disorders	Generalized cost-effectiveness analysis for national-level priority-setting in the health sector	Field testing of a multicriteria decision analysis (MCDA) framework for coverage of a screening test for cervical cancer in South Africa	Purchasing of medical equipment in public hospitals: the mini-HTA tool	Equity-oriented toolkit for health technology assessment and knowledge translation: application to scaling up of training and education for health workers
**Ease of training**	Yes, it is an easy to use tool with clear guidelines and criteria	Yes, it is an easy to use tool with clear instructions and would require little training	No, special training required	Yes, not a difficult approach	Yes, the mini-HTA tool is easy to use and the questions on the form are clear	Yes, not a difficult approach
**Is the evidence collection requirement flexible?**	Yes, it allows the use of less systematic evidence gathering with justification	Yes, all evidence in the form of data about medical devices is provided	Yes, data to assess the effectiveness of a medical technology not only come from reviews of evidence, but population surveys and expert opinion	Yes, available data in local context is used to customize framework	Yes, available data in the local context is used	No, only mentions sources such as The Cochrane Library and it is unclear if all other sources are considered
**Is approach or tool economical?**	Yes, does not require expensive equipment or services	Yes, user would only need a computer and the software	Yes, does not require expensive equipment or services	Yes, does not require expensive equipment or services	Yes, tool only comprises of one form and not expensive equipment or services are required	Yes, does not require expensive equipment or services
**Does approach or tool address local context, including political framework?**	Yes, it considers the local context throughout its process	No, the tool itself does not consider the local context	Yes, modifications according to local context is recommended by approach	Yes, HTA report is tailored to reflect local context	Yes, it is flexible and adapted to fit the local context	Yes, involves stakeholders in the process which bring in a contextual perspective
**Considerations**	Does not include an explicit priority setting system. However, this tool is used to assess specific health technologies so it is further down in the HTA process. Evidence has the potential of not being very robust	Economical standing of this tool is dependent on the price of the decision making assistance software	Economical standing of this approach is dependent of amount of special training needed		The mini-HTA tool is a fast tool; however, it is not very comprehensive and only vaguely addresses the principles that it proposes. In addition, one of its limitations is that it is only applied in the context of hospital settings	

## Results

**Figure 1:**
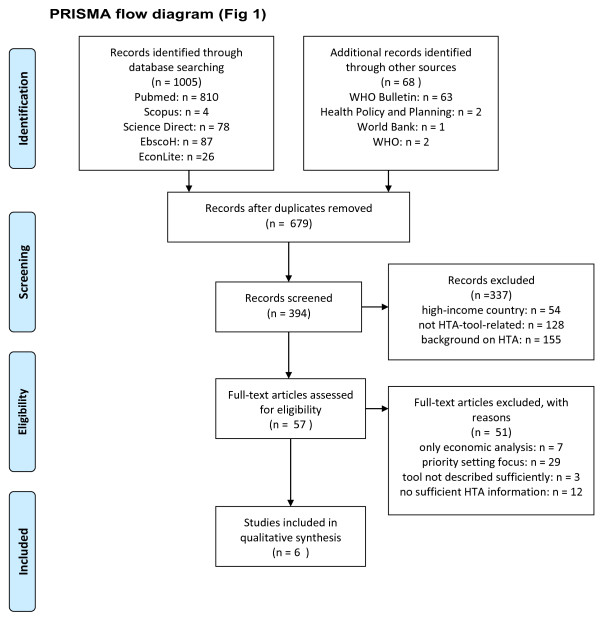
**PRISMA flow diagram.**

The search retrieved 1,073 papers in total, of which six fulfilled the inclusion criteria (PRISMA flowchart, Figure 1). Data was extracted from the six papers published between 2003 and 2013.

HTA tools overview:

-  WHO CHOICE cost-effectiveness analysis tool [13]
-  KNOW ESSENTIALS tool using thirteen criteria related to context-specific HTA and prioritization of these criteria [8]
-  Weighting of Multi-Criteria Decision Analysis criteria on the basis of corresponding HTA report [14]
-  Adaptation of Mini-HTA or hospital-based HTA tool for the decision-making related to medical devices purchased in a hospital setting [15]
-  Equity-oriented toolkit for HTA, containing four elements: burden of illness, community effectiveness, economic evaluation and knowledge translation [16]
-  Mapping system using Analytic Hierarch Process (AHP) methods between different diseases and their matching technologies to minimize technology underutilization [17]

None of the papers were funded or stated funding by other sources. Four studies proposed novel tools for HTA [8, 15–17] and the other two used current HTA approaches originally developed for high-income settings [13, 14]. One study specified their target population to be in SSA countries [15], the others focused on “resource-poor” settings [13, 14, 16, 17] and settings without formal HTA [8]. However, all studies included in the review can be applied in the SSA setting. From the proposed tools, two were stand-alone [8, 16] and the other two were support tools [15, 17]. The support tools served as decision support in the purchase and acquisition of cardiovascular disorder equipment [17] and the scaling up of training and education of health workers [16]. The current HTA approaches used in the studies were: multi-criteria decision analysis (MCDA) [14], the WHO-CHOICE project [13], and generalized cost-effectiveness analysis [13]. Four papers applied their tool or approach [8, 14, 15, 17], two of those were pilot tests [8, 17]. The other two approaches and tools were not applied and only described proposed HTA approaches or tools [13, 16].

Two of the tools were focused on pharmaceuticals [8, 13], whereas three HTA tools were focused on medical devices [14, 15, 17]. For the medical devices, varying contexts were chosen: one study focused on the introduction of a screening test [14], another on the underutilisation of medical devices [17] and a third study focused on the decision-making process for the purchasing of medical devices [15]. One tool was centred on a health education intervention [16].

### Structure of HTA programs

All of the papers were found to explicitly address relevant goals and scopes of their HTA tool or approach. Five studies were unbiased and had transparent processes [8, 13, 14, 16, 17]. One tool required subjective assessment during its process [15]. Five methods included all relevant technologies by considering all alternatives [8, 14, 15, 17] or by using an extensive database to do so [13], while the sixth study did not state if this was addressed [16].

### Methods of HTA

A wide range of evidence and outcomes was considered by all studies [8, 13–17] and appropriate methods for assessing costs and benefits were taken up by five approaches [8, 13–16]. One study did not currently assess the costs [17]. Three considered a full societal perspective [8, 15, 16], whereas the other three failed to state this [13, 14, 17]. Only three of the tools or approaches stated explicitly characterizing uncertainty surrounding their estimates [13–15]. Issues of generalizability and transferability are addressed by four of the papers [8, 13, 14, 16].

### Processes for conducting HTA

All of the approaches required for all available data to be sought [8, 13–17]. Two of the papers failed to actively include all key stakeholders [13, 17], whereas all others included key stakeholders by including them in the process or considering them throughout [8, 14–16]. How findings would be monitored was not stated in any of the papers.

### Use of HTA in decision-making

Only two studies mentioned a timeframe for completing the HTA, which also was timely [15, 17]. Findings were communicated appropriately to different decision makers by four tools or approaches [8, 14–16]. Three of the papers clearly defined the link between HTA findings and decision-making processes [8, 14, 15], while the others did not state this [13, 15, 17].

### Principles for HTA activities specific for SSA

In the context of SSA, five papers presented tools or approaches that were easy to use [8, 14–17], whereas one required special training to carry out the generalized cost-effectiveness analysis [13]. The evidence requirement was flexible and included available data whether from a literature search or other less rigorous, yet justified, sources when it was not available for five of the studies [8, 13–15, 17]. One was not as flexible by requiring the data to be collected in databases such as the Cochrane Library and no additional alternate sources were mentioned [16]. Only one of the papers failed to address the local context explicitly in their tool [17].

## Discussion

### HTA advantages and areas of use

The advantages of using HTA are first and foremost the systematic evaluation of cost and effectiveness of medical technologies and allowing health systems to achieve the greatest good for the greatest number of patients. Chalkidou et al. highlight the importance of HTA for universal health coverage systems by efficiently and equitably allocating resources [18]. This focused resource use has an effect on better budgeting and long-term financial sustainability of the health systems in SSA countries. It is crucial that the increased use of HTA in these countries is complemented by capacity building and increased expertise in the HTA area in order to ensure a sustainable infrastructure [19]. International collaboration among HTA bodies can facilitate the development of methods and more efficient assessment processes, and facilitate knowledge transfer and capacity-building in less established HTA systems and programmes.

Another area of need is emerging for relevant applications of HTA, namely its use in global funding organisations as a means for increased value for money. Teerawattananon et al. have argued for the use of HTA approaches for the Global Fund to Fight HIV/AIDS, Tuberculosis and Malaria in order to provide strategic directions for the prioritization of health care interventions currently funded by the organization [20]. Other global health initiatives and national donor countries may follow suit in conducting HTAs before, during, and after grant implementation in order to improve efficiency and identify areas of unmet need (in June 2014 the Gates Reference Case has been launched which involves more principled cost-effectiveness analysis in health programme funding by the Bill and Melinda Gates Foundation). Therefore, the necessity for the thorough evaluation and fine-tuning of relevant HTA tools and approaches becomes more evident.

### Lack of application

The review shows widespread methodological heterogeneity among the different studies included in the review. The HTA tools or approaches used varied a lot in their context and scope. There was a lack of application of some tools for a specific medical technology or intervention in the SSA setting. The tools analysed in this review would benefit from a wider application and pilot-testing as well as user friendliness. A direct comparison between applying the tools or approaches would also highlight the advantages and disadvantages of each tool or approach and guide decision makers in regards to which tools should be used, in which context, and for which tasks.

### Robustness of HTA

While the need for HTA in SSA is evident, the robustness of HTA cannot be neglected when resources are limited. The majority of the approaches involved a well-rounded structure for their HTA that was accomplished with explicit goals and scopes, unbiased and transparent processes, and the inclusion of alternative technologies and some also had priority setting processes incorporated [14, 16, 17]. In this context, “priority-setting” focuses on identifying different health technologies for which an evaluation regarding their inclusion in the health system is warranted, while HTA relates to the actual evaluation of a specific technology.

The methods utilized in the approaches followed the principles for HTA activities in most of the studies. However, one approach that seemed to be limited by its purpose as a computerized decision-making aide [17] seemed to lack robustness in its methods by not assessing costs. The processes for conducting HTA were limited for some approaches by the exclusion of key stakeholders [13, 17], an integral dimension of good practice in HTA methodology. In addition, the monitoring of HTA practices lacked in all of the studies. When assessing the use of HTA in decision-making, two approaches showed limitations [13, 17]. One approach completely lacked the inclusion of the principles in this section, but this may be connected with the priority setting orientation of the approach [13]. All of the tools addressed the issue of economic resource deficiency; however, they lacked specifications explicitly considering the local context [17], requiring little training for their use [13] and being flexible with the data necessary for undertaking the HTA when there is limited data or access available [16].

### Potential opportunities

In general, the review revealed that the majority of approaches were not applied in a stand-alone manner and were rather used as a support tool to existing decision-making processes. The analysis revealed that two approaches show particular promise for further investigation: the KNOW ESSENTIAL tool for its compact, yet comprehensive coverage [8], and the MCDA approach for the active involvement of stakeholders in its process [14]. These incorporated all aspects important for the SSA context into their evaluation such as contextual issues, flexible data collection practices, and economical and easy use. However, it is important to point out that the MCDA approach is, per se, focused on assessing available evidence and not generating new evidence as in the case with other HTA tools. Although several MCDA tools have been applied in the SSA setting, we only included one study in our review as it was explicitly connected with the assessing of evidence in the context of a HTA report.

Even though the mini-HTA tool [15] also incorporated the majority of the principles for HTA activities and all of the contextual SSA principles, it had a number of limitations related to comprehensive and detailed coverage due to its restriction to the hospital setting [15]. The critical appraisal also revealed that other tools did not meet all of the contextual criteria for SSA [13, 16, 17] or were limited to a position further down-stream in the decision making process. Although it is possible to weight the criteria applied in the KNOW ESSENTIALS tool, it would be beneficial and interesting to combine the tool with aspects of MCDA in order to allow for a more detailed stakeholder evaluation and prioritization of the evidence.

A thorough evaluation of the available tools is highly desirable, involving a wide range of academic and ministry of health partners in a SSA setting in order to ensure context-specific application of HTA tools. Specific emphasis should be made on the need for HTA evaluations to allow for differences in the evaluations of pharmaceuticals and medical devices. The majority of HTAs currently focus on the assessment of pharmaceuticals and tend to neglect medical devices. Due to the varying focus of the HTA tools that are centred on the evaluation of medical devices in our review, it is not possible to draw clear conclusions on the appropriate emphasis of the development of medical device HTA tools.

### Study limitations

The methodology for the evaluation on each quality criteria point highlighted in Table 3 “Extraction Form for Principles for HTA Activities in SSA” is based on broad expert opinion elicitation in our research consortium, which represents different areas and institutions such as academia, WHO, HTA expertise, and political decision makers. Ultimately, the evaluation on the basis of expert opinion is a subjective assessment of the research consortium which may be subject to potential bias and as such has to be highlighted as a study limitation.

## Conclusions

Our review has emphasised that there is a lack of HTA tools that can be used for systematically assessing technology in the SSA context. A clear gap in HTA methodology focused on “resource-limited” settings, and particularly the SSA context, calls for more research into further evaluating and developing relevant HTA methods and approaches, especially in the context of the WHO resolution on “Health Intervention and Technology Assessment in Support of Universal Health Coverage”.

An appropriate HTA tool for “resource-constrained” settings, and especially SSA, should address all important criteria of decision making. By combining the two most promising tools, KNOW ESSENTIALS and MCDA, appropriate analysis of evidence with a robust and flexible methodology could be applied for the SSA setting. Although there are a range of arguments favouring the need for HTA in the SSA context, advocacy for the importance of HTA in these settings needs to emerge more clearly [18].

## Appendix

### Search string

Keywords included synonyms for the following topic (“Health Technology Assessment” OR “HTA” OR “health technology evaluation” OR “priority setting”) AND (“Developing countr*” OR “low income countr*” OR “resource-limited” OR “resource-constrain” OR “Africa”). In addition, the names of all sub-Saharan African countries were separately listed as search terms:

“Developing countr*” OR “low income countr*” OR “resource-limited” OR “resource-constrain*” OR Africa* OR Angola OR Benin OR Botswana OR “Burkina Faso” OR Burundi OR Cameroon OR “Cape Verde” OR “Central African Republic” OR Chad OR Comoros OR Congo OR “Democratic Republic of Congo” OR Djibouti OR “Equatorial Guinea” OR Eritrea OR Ethiopia OR Gabon OR Gambia OR Ghana OR Guinea OR “Guinea Bissau” OR “Ivory Coast” OR “Cote d’Ivoire” OR Jamahiriya OR Jamahiryia OR Kenya OR Lesotho OR Liberia OR Madagascar OR Malawi OR Mali OR Mauritania OR Mauritius OR Mayote OR Mozambique OR Mocambique OR Namibia OR Niger OR Nigeria OR Principe OR Reunion OR Rwanda OR “Sao Tome” OR Senegal OR Seychelles OR “Sierra Leone” OR Somalia OR “South Africa” OR “St Helena” OR Sudan OR Swaziland OR Tanzania OR Togo OR Tunisia OR Uganda OR “Western Sahara” OR Zambia OR Zimbabwe AND “Health Technology Assessment” OR HTA OR “health technology evaluation” OR “priority setting”.

## Abbreviations

HTA: Health technology assessment
MCDA: Multi-criteria decision analysis
SSA: Sub-Saharan Africa.
